# Comparative efficacy of subcutaneous (CT-P13) and intravenous infliximab in adult patients with rheumatoid arthritis: a network meta-regression of individual patient data from two randomised trials

**DOI:** 10.1186/s13075-021-02487-x

**Published:** 2021-04-16

**Authors:** Bernard Combe, Yannick Allanore, Rieke Alten, Roberto Caporali, Patrick Durez, Florenzo Iannone, Michael T. Nurmohamed, Mondher Toumi, Sang Joon Lee, Taek Sang Kwon, Jiwon Noh, Gahee Park, Dae Hyun Yoo

**Affiliations:** 1 Department of Rheumatology, CHU Montpellier, Montpellier University, Montpellier, France; 2 Rheumatology Department, Hôpital Cochin, Paris Descartes University, Paris, France; 3 Department of Internal Medicine and Rheumatology, Schlosspark-Klinik Charité, University Medicine Berlin, Berlin, Germany; 4 Department of Clinical Sciences and Community Health, Research Center for Adult and Pediatric Rheumatic Diseases, University of Milan, Milan, Italy; 5 ASST PINI-CTO, Milan, Italy; 6 Rheumatology, Cliniques Universitaires Saint-Luc – Universite Catholique De Louvain – Institut De Recherche Experimentale Et Clinique (IREC), Brussels, Belgium; 7 Rheumatology Unit, Department of Emergency and Organ Transplantation, Università Degli Studi Di Bari Aldo Moro, Bari, Italy; 8 Department of Rheumatology, Amsterdam Rheumatology and Immunology Center, Reade, Amsterdam, The Netherlands; 9 Department of Rheumatology, Amsterdam Rheumatology and Immunology Center, VU University Medical Center, Amsterdam, The Netherlands; 10 Department of Public Health, Aix-Marseille University, Marseille, France; 11 Celltrion Inc., Incheon, Republic of Korea; 12 Celltrion Healthcare Co., Ltd, Incheon, Republic of Korea; 13 Department of Rheumatology, Hanyang University Hospital for Rheumatic Diseases, Seoul, Republic of Korea

**Keywords:** CT-P13, Disease activity, Indirect treatment comparison, Individual patient data, Infliximab, Intravenous, Network meta-regression, Rheumatoid arthritis, Subcutaneous, Tumour necrosis factor inhibitor

## Abstract

**Background:**

A subcutaneous (SC) formulation of infliximab biosimilar CT-P13 is approved in Europe for the treatment of adult patients with rheumatoid arthritis (RA). It may offer improved efficacy versus intravenous (IV) infliximab formulations.

**Methods:**

A network meta-regression was conducted using individual patient data from two randomised trials in patients with RA, which compared CT-P13 SC with CT-P13 IV, and CT-P13 IV with reference infliximab IV. In this analysis, CT-P13 SC was compared with CT-P13 IV, reference infliximab IV and pooled data for both reference infliximab IV and CT-P13 IV. Outcomes included changes from baseline in 28-joint Disease Activity Score based on C-reactive protein (DAS28-CRP), Simplified Disease Activity Index (SDAI) and Clinical Disease Activity Index (CDAI), and rates of remission, low disease activity or clinically meaningful improvement in functional disability per Health Assessment Questionnaire–Disability Index (HAQ-DI).

**Results:**

The two studies enrolled 949 patients with RA; pooled data for 840 and 751 patients were evaluable at weeks 30 and 54, respectively. For the CT-P13 SC versus pooled IV treatment arm comparison, differences in changes from baseline in DAS28-CRP (− 0.578; 95% confidence interval [CI] − 0.831, − 0.325; *p* < 0.0001), CDAI (− 3.502; 95% CI − 5.715, − 1.289; *p* = 0.002) and SDAI (− 4.031; 95% CI − 6.385, − 1.677; *p* = 0.0008) scores at 30 weeks were statistically significant in favour of CT-P13 SC. From weeks 30 to 54, the magnitude of the differences increased and remained statistically significant in favour of CT-P13 SC. Similar results were observed for the comparison of CT-P13 SC with CT-P13 IV and with reference infliximab IV. Statistically significant differences at week 30 favoured CT-P13 SC over the pooled IV treatment arms for the proportions of patients achieving EULAR-CRP good response, American College of Rheumatology (ACR) 50 and ACR70 responses, DAS28-CRP-defined remission, low disease activity (DAS28-CRP, CDAI and SDAI criteria) and clinically meaningful HAQ-DI improvement.

**Conclusions:**

CT-P13 SC was associated with greater improvements in DAS28-CRP, CDAI and SDAI scores and higher rates of clinical response, low disease activity and clinically meaningful improvement in functional disability, compared with CT-P13 IV and reference infliximab IV.

**Trial registration:**

EudraCT, 2016-002125-11, registered 1 July 2016; EudraCT 2010-018646-31, registered 23 June 2010.

**Supplementary Information:**

The online version contains supplementary material available at 10.1186/s13075-021-02487-x.

## Background

Rheumatoid arthritis (RA) is a systemic inflammatory autoimmune disease that affects over 3.0 million people in Europe [1]. Symptoms include joint pain, swelling and stiffness, potentially leading to joint damage and irreversible disability [2–5]. Patients with RA have higher rates of disability than the general population [6], and many patients experience reduced work productivity and health-related quality of life (HRQoL) [7, 8].

Optimal use of medication is a key strategy for effective RA management [9]. The primary treatment target for patients with RA is sustained remission, with low disease activity as an alternative target, particularly for patients with long-standing disease [9, 10]. European League Against Rheumatism (EULAR) recommendations advocate the initiation of therapy with disease-modifying antirheumatic drugs (DMARDs) as soon as a diagnosis of RA is made [9]. The addition of a biologic DMARD (bDMARD) or targeted synthetic DMARD (tsDMARDs) is recommended when treatment targets are not achieved with the first conventional synthetic DMARD (csDMARD) and poor prognostic factors are present [9].

Tumour necrosis factor inhibitors (TNFis) are a class of bDMARD that are well tolerated and have been shown to effectively reduce disease activity and structural joint damage [9, 11]. Five TNFis are currently approved for the treatment of RA (infliximab, adalimumab, etanercept, golimumab and certolizumab pegol) [9]. Most of these are administered subcutaneously (SC); only reference infliximab intravenous (IV; Remicade®, Janssen Biologics BV) and biosimilars of infliximab are administered intravenously (although the feasibility of SC administration was reported as early as 2006 [12]). CT-P13 IV (Remsima® IV, Celltrion Healthcare Co., Ltd.), an infliximab biosimilar, received European Union (EU) authorisation for the treatment of adult patients with RA in 2013 [13] and US approval in 2016 [14]. Subsequently, CT-P13 SC (Remsima® SC, Celltrion Healthcare Co., Ltd), the only SC formulation of infliximab, received EU approval in 2019 [13, 15]. For maintenance therapy in patients with RA, the approved doses for CT-P13 IV and CT-P13 SC are 3 mg/kg every 8 weeks (Q8W) and 120 mg every 2 weeks (Q2W), respectively [13].

CT-P13 SC and CT-P13 IV were approved based on the results of the pivotal 3.5 (NCT03147248; EudraCT No. 2016-002125-11) and 3.1 (NCT01217086; EudraCT No. 2010-018646-31) trials, respectively. The CT-P13 3.5 trial was a randomised, multicentre, parallel-group, phase I/III study that enrolled 357 patients with active RA [16]. In this study, non-inferiority of CT-P13 SC to CT-P13 IV was demonstrated using assessment of the change from baseline in the 28-joint Disease Activity Score based on C-reactive protein (DAS28-CRP) at week 22, with a statistically significant treatment difference of 0.27 (95% confidence interval [CI] 0.02, 0.52) for the SC versus the IV treatment arm, although the 95% CI was higher than the predefined non-inferiority margin of − 0.06. Other efficacy outcomes were generally comparable between the SC and IV treatment arms up to week 22 and favoured the SC arm at week 30 [16]. The CT-P13 3.1 trial was a randomised, double-blind, parallel-group, phase III study of 606 patients with RA [17, 18]. In this study, therapeutic equivalence was established between CT-P13 IV and reference infliximab IV at week 30 [17], and similar efficacy and safety profiles were demonstrated up to week 54 [18].

To date, there have been no head-to-head comparisons of CT-P13 SC versus reference infliximab IV, and the comparison of CT-P13 SC versus CT-P13 IV was limited to 30 weeks as all patients randomised to CT-P13 IV in the 3.5 trial were switched to CT-P13 SC at week 30. However, the availability of patient-level data from the CT-P13 3.1 and 3.5 trials allows a mixed treatment comparison of CT-P13 SC versus infliximab IV at weeks 30 and 54. The objective of this analysis was to examine whether CT-P13 SC provides added clinical value compared with CT-P13 IV and reference infliximab IV using individual patient data (IPD) from the CT-P13 3.1 and 3.5 trials.

## Methods

### Data sources

Data were sourced from two randomised controlled trials (RCTs): the CT-P13 3.1 trial, which compared CT-P13 IV with reference infliximab IV [17, 18], and the CT-P13 3.5 trial, which compared CT-P13 IV with CT-P13 SC [16]. In both trials, patients received a similar dose of methotrexate at 12.5–25 mg/week (along with folic acid at a dose of ≥ 5 mg/week), which was maintained throughout the study [16, 17]. At baseline in the CT-P13 3.1 trial, the mean (standard deviation [SD]) methotrexate dose was 15.6 (3.1) and 15.6 (3.2) mg for patients in the CT-P13 IV and reference infliximab IV treatment arms, respectively [17]. In the CT-P13 3.5 trial, the mean (SD) methotrexate dose at baseline was 17.0 (4.0) and 17.4 (4.0) mg/week in the CT-P13 SC and CT-P13 IV treatment arms, respectively [16]. IPD, including patient characteristics and outcomes, were sourced from both trials.

The CT-P13 3.1 and 3.5 clinical trials both included a CT-P13 IV treatment arm, permitting indirect comparison of CT-P13 SC with either reference infliximab IV or with pooled data for reference infliximab IV and CT-P13 IV, at week 30.

### Treatment comparisons

CT-P13 SC was compared to (1) CT-P13 IV (from studies 3.1 and 3.5), (2) reference infliximab IV (from study 3.1) and (3) pooled data including both reference infliximab IV and CT-P13 IV (from studies 3.1 and 3.5).

### Efficacy outcomes of interest

Efficacy outcomes included in the present analysis were the change from baseline in DAS28-CRP [19]; change from baseline in Simplified Disease Activity Index (SDAI) [20]; change from baseline in Clinical Disease Activity Index (CDAI) [20]; remission based on SDAI and CDAI, Boolean remission (TJC ≤ 1, SJC ≤ 1, CRP ≤ 1 mg/dL and PGA ≤ 1 at any time point), and remission also defined as DAS-28-CRP (< 2.6) [21]; low disease activity based on DAS28-CRP (≤ 3.2), CDAI (≤ 10.0) and SDAI (≤ 11.0) [22]; EULAR response [23]; and American College of Rheumatology (ACR) response (ACR20, ACR50 and ACR70) [24, 25]. Additionally, functional disability was assessed based on the proportion of patients with a change in Health Assessment Questionnaire–Disability Index (HAQ-DI) that was equal to or greater than the minimal clinically important difference (MCID) of 0.22 [26, 27].

### Feasibility assessment

The studies contributing data to the present analyses had similar eligibility criteria and the same target population. Definitions of all outcomes of interest were also the same. Baseline demographics (e.g. age, sex, body mass index) were similar between studies, although a higher proportion of Asian patients were enrolled in the 3.1 trial versus the 3.5 trial (Table 1). Baseline clinical characteristics were also similar in terms of objectively measured parameters, such as CRP, erythrocyte sedimentation rate (ESR), DAS28-CRP, 28-joint Disease Activity Score based on ESR (DAS28-ESR), Swollen 28-Joint Count (SJC) and Tender 28-Joint Count (TJC) (Table 1). Small differences were observed in patients’ and physicians’ global assessment of disease activity; these measures were numerically greater in the 3.5 trial, compared to the 3.1 trial (Table 1).

**Table 1:** Summary of patient characteristics at baseline in studies 3.1 and 3.5

Variable		Study 3.1			Study 3.5		
		Reference IFX IV (*n* = 304)	CT-P13 IV (*n* = 302)	All (*n* = 606)	CT-P13 IV (*n* = 176)	CT-P13 SC (*n* = 167)	All (*n* = 343)
**Demographics**							
Sex	Male, *n* (%)	48 (15.8%)	57 (18.9%)	105 (17.3%)	37 (21.0%)	37 (22.2%)	74 (21.6%)
	Female, *n* (%)	256 (84.2%)	245 (81.1%)	501 (82.7%)	139 (79.0%)	130 (77.8%)	269 (78.4%)
Race	White, *n* (%)	222 (73.0%)	220 (72.8%)	442 (72.9%)	151 (85.8%)	145 (86.8%)	296 (86.3%)
	Asian, *n* (%)	37 (12.2%)	34 (11.3%)	71 (11.7%)	2 (1.1%)	1 (0.6%)	3 (0.9%)
	Black, *n* (%)	1 (0.3%)	2 (0.7%)	3 (0.5%)	–	–	–
	Other, *n* (%)	44 (14.5%)	46 (15.2%)	90 (14.9%)	23 (13.1%)	21 (12.6%)	44 (12.8%)
Region	European, *n* (%)	199 (65.5%)	196 (64.9%)	395 (65.2%)	147 (83.5%)	141 (84.4%)	288 (84.0%)
	Non-European, *n* (%)	105 (34.5%)	106 (35.1%)	211 (34.8%)	29 (16.5%)	26 (15.6%)	55 (16.0%)
Age, years	Mean ± SD	48.6 ± 11.5	49.0 ± 12.2	48.8 ± 11.8	51.9 ± 12.4	50.9 ± 12.2	51.4 ± 12.3
Baseline weight, kg	Mean ± SD	69.9 ± 15.8	70.7 ± 16.3	70.3 ± 16.0	72.7 ± 14.4	73.0 ± 15.1	72.9 ± 14.7
Baseline BMI, kg/m^2^	Mean ± SD	26.3 ± 5.3	26.5 ± 5.3	26.4 ± 5.3	26.8 ± 4.1	26.8 ± 4.4	26.8 ± 4.3
**Clinical characteristics**							
CRP, mg/L	Mean ± SD	18.9 ± 21.8	19.0 ± 25.1	18.9 ± 23.5	22.4 ± 35.4	18.3 ± 23.7	20.4 ± 30.2
ESR, mm/h	Mean ± SD	48.5 ± 22.6	46.5 ± 22.3	47.5 ± 22.4	44.6 ± 23.5	41.8 ± 19.1	43.2 ± 21.5
DAS28-CRP	Mean ± SD	5.8 ± 0.9	5.9 ± 0.8	5.8 ± 0.9	5.9 ± 0.8	6.0 ± 0.8	5.9 ± 0.8
DAS28-ESR	Mean ± SD	6.6 ± 0.8	6.7 ± 0.8	6.6 ± 0.8	6.6 ± 0.8	6.7 ± 0.8	6.6 ± 0.8
CDAI	Mean ± SD	39.4 ± 11.0	40.9 ± 11.4	40.2 ± 11.2	39.6 ± 10.0	42.7 ± 10.2	41.1 ± 10.2
SDAI	Mean ± SD	41.3 ± 11.6	42.8 ± 11.9	42.1 ± 11.8	41.9 ± 11.1	44.5 ± 10.7	43.1 ± 11.0
SF-36 Mental	Mean ± SD	37.8 ± 11.1	36.8 ± 10.7	37.3 ± 10.9	39.6 ± 10.5	39.9 ± 10.3	39.8 ± 10.4
SF-36 Physical	Mean ± SD	31.9 ± 7.2	31.1 ± 6.1	31.5 ± 6.7	33.3 ± 6.3	33.6 ± 5.6	33.5 ± 6.0
Patient assessment of global disease activity	Mean ± SD	65.4 ± 17.0	65.8 ± 17.2	65.6 ± 17.1	69.0 ± 17.5	70.5 ± 15.8	69.7 ± 16.7
Physician assessment of global disease activity	Mean ± SD	65.0 ± 13.5	64.9 ± 14.2	64.9 ± 13.8	68.9 ± 15.2	70.5 ± 14.1	69.7 ± 14.7
HAQ-DI	Mean ± SD	1.6 ± 0.6	1.6 ± 0.5	1.6 ± 0.6	1.6 ± 0.6	1.6 ± 0.5	1.6 ± 0.6

*BMI* body mass index, *CDAI* Clinical Disease Activity Index, *CRP* C-reactive protein, *DAS28-CRP* 28-joint Disease Activity Score based on C-reactive protein, *DAS28-ESR* 28-joint Disease Activity Score based on erythrocyte sedimentation rate, *ESR* erythrocyte sedimentation rate, *HAQ-DI* Health Assessment Questionnaire–Disability Index, *IFX* infliximab, *IV* intravenous, *SC* subcutaneous, *SDAI* Simplified Disease Activity Index, *SD* standard deviation

### Statistical analyses

Baseline participant characteristics were presented using descriptive statistics: mean ± SD for continuous variables and percentages for categorical variables. All statistical analyses were conducted using SAS statistical software (version 9.3, SAS Institute, Cary, USA). Two-sided tests were used and *p*-values < 0.05 were considered statistically significant.

#### Data imputation at week 54

**Fig. 1:**
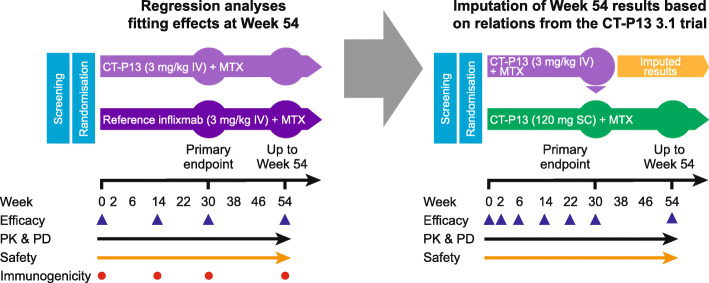
Imputation of week-54 CT-P13 3.5 results based on CT-P13 regression model. EU, European Union; IV, intravenous; MTX, methotrexate; PD, pharmacodynamics; PK, pharmacokinetics

Because all participants randomised to CT-P13 IV in the 3.5 trial switched to CT-P13 SC at week 30, week 54 effects in the CT-P13 IV treatment arm were imputed using regression methods based on the effects observed in the CT-P13 IV treatment arm of the CT-P13 3.1 trial (Fig. 1). Linear regression models were fitted using IPD from the CT-P13 IV treatment arm of the CT-P13 3.1 trial. The dependent variables were the changes from baseline to week 54 in DAS28-CRP, CDAI and SDAI, respectively. The model covariates were the values of the modelled outcome at baseline and the change from baseline to week 30, as well as possible confounders selected from a list shown in Additional file 1, Table S1. The selection of potential confounders was performed in three steps: (1) the association of each variable listed in Table S1 with the change from baseline to week 54 in modelled outcome was tested; (2) if several variables were correlated (Pearson *r* ≥ 0.6 for continuous variables; see correlation coefficients in Additional file 1, Table S2), only the variable with the strongest association with the modelled outcomes was retained; and (3) all selected variables from step 2 were entered in the model and a backward selection procedure was applied to further reduce the list of variables.

*R*^2^ was used to assess the quality of the models. *R*^2^ was 0.48 for the model predicting change from baseline in DAS28-CRP score and 0.61 for the models predicting the changes from baseline in CDAI and SDAI, demonstrating that the quality of models was acceptable. The models are presented in Additional file 1, Table S3.

The obtained regression models provided predictions of the mean changes from baseline to week 54 in DAS28-CRP, CDAI and SDAI and associated SDs. A multiple imputation method was used to account for the uncertainty around the predicted values [28]. For each patient, 10 values were generated randomly from the statistical distribution around the predicted scores, thus generating 10 datasets on which the meta-regression models were estimated. Imputation was not performed for binary outcomes because prediction of the outcome itself was surrounded with a large degree of uncertainty.

#### Meta-regression

A network meta-regression using IPD is recommended as the “gold standard” method to adjust for treatment effect modifiers when IPD are available for all considered studies [29, 30]; this method was implemented here in accordance with relevant methodological guidelines [29].

Two series of analyses were performed, using two definitions of the treatment variable: treatment variable with three levels (CT-P13 SC, CT-P13 IV, reference infliximab IV) and treatment variable with two levels (CT-P13 SC, infliximab IV [pooled data for CT-P13 IV and reference infliximab IV]).

Multivariate mixed models, with normal distribution and identity link function for continuous outcomes and binomial distribution and logit link function for binary outcomes, were fitted at weeks 30 and 54. Dependent variables were the outcomes of interest, as listed above. Model covariates were selected among variables listed in Additional file 1, Table S1. The same 3-step approach as described for the imputation model above was used for the models for DAS28-CRP, CDAI and SDAI change from baseline, EULAR good response (CRP criteria), ACR20, ACR50, ACR70, Boolean remission and HAQ-DI MCID (≥ 0.22) at week 30. For binary outcomes based on DAS28-CRP, CDAI and SDAI, the same covariates as in the model for corresponding continuous outcomes were used. For outcomes at week 54, the same covariates as for the corresponding outcomes at week 30 were used. In addition, a variable representing study 3.1 or 3.5 was entered in all regression models as a random effect.

Analysis outputs included treatment differences with associated 95% CIs for continuous outcomes, and odd ratios (OR) with associated 95% CIs for binary outcomes.

The treatment effect at week 54, for each continuous outcome (DAS28-CRP, CDAI and SDAI), was obtained as the mean of treatment effects estimated from the 10 simulated datasets, and the associated variance coefficient was calculated as the sum of the variance of estimated treatment effect within simulations and variance between simulations [28, 31]. *p*-value and 95% CI calculations considered a normal distribution of finally obtained coefficients.

## Results

### Mixed treatment comparison at week 30

**Fig. 2:**
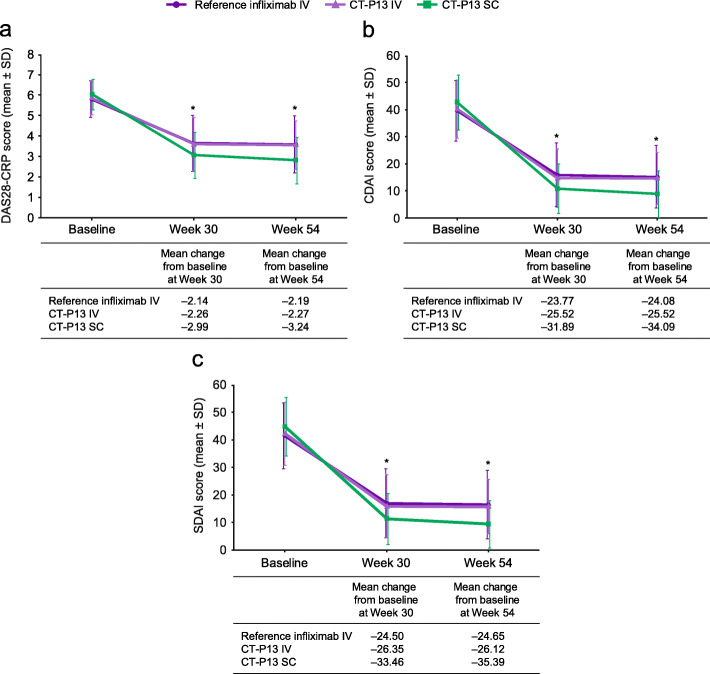
Change from baseline in DAS28-CRP (**a**), CDAI (**b**) and SDAI (**c**) at weeks 30/54. CDAI, Clinical Disease Activity Index; CRP, C-reactive protein; DAS28-CRP, 28-joint Disease Activity Score based on C-reactive protein; IV, intravenous; SDAI, Simple Disease Activity Index; SC, subcutaneous; SD, standard deviation. *CT-P13 IV vs CT-P13 SC *p* < 0.05, CT-P13 SC vs reference infliximab IV *p* < 0.05 and CT-P13 IV vs reference infliximab IV *p* ≥ 0.05. *p*-values derive from the meta-regression efficacy analysis of week-30 and week-54 data (Tables 3 and 4)

Figure 2 presents pooled data for unadjusted estimates of outcomes at weeks 30 and 54, according to treatment. Table 2 presents pooled data for unadjusted response and remission rates at week 30, according to treatment.

**Table 2:** Response, remission and low disease activity rates by treatment arm at week 30 (pooled data)

*n/N* (*%*)	Reference IFX IV	CT-P13 IV	CT-P13 SC
Patients achieving a EULAR good response (CRP criteria)	98/259 (37.8%)	165/415 (39.8%)	83/157 (52.9%)
Patients achieving ACR20 response	179/261 (68.6%)	318/418 (76.1%)	142/161 (88.2%)
Patients achieving ACR50 response	103/261 (39.5%)	195/418 (46.7%)	106/161 (65.8%)
Patients achieving ACR70 response	47/261 (18.0%)	97/418 (23.2%)	68/161 (42.2%)
DAS28-CRP			
Patients achieving low disease activity: DAS28-CRP ≤ 3.2	101/259 (39.0%)	166/415 (40.0%)	84/157 (53.5%)
Patients achieving remission: DAS28-CRP < 2.6	60/259 (23.2%)	101/415 (24.3%)	59/157 (37.6%)
CDAI			
Patients achieving low disease activity: CDAI ≤ 10.0	93/259 (35.9%)	172/416 (41.3%)	92/161 (57.1%)
Patients achieving remission: CDAI ≤ 2.8	22/259 (8.5%)	50/416 (12.0%)	27/161 (16.8%)
SDAI			
Patients achieving low disease activity: SDAI ≤ 11.0	96/259 (37.1%)	172/415 (41.4%)	89/157 (56.7%)
Patients achieving remission: SDAI ≤ 3.3	22/259 (8.5%)	53/415 (12.8%)	29/157 (18.5%)
Patients achieving Boolean remission	6/260 (2.3%)	12/416 (2.9%)	12/161 (7.5%)
Patients achieving HAQ-DI MCID (≥ 0.22)	15/261 (5.7%)	36/418 (8.6%)	30/161 (18.6%)

*ACR* American College of Rheumatology, *CDAI* Clinical Disease Activity Index, *CRP* C-reactive protein, *DAS28-CRP* 28-joint Disease Activity Score based on C-reactive protein, *EULAR* European League Against Rheumatism, *HAQ-DI* Health Assessment Questionnaire–Disability Index, *IFX* infliximab, *IV* intravenous, *MCID* minimal clinically important difference, *SC* subcutaneous, *SDAI* Simple Disease Activity Index

A total of 840 patients were included in the meta-regression efficacy analysis of week 30 data (Table 3). For the comparison between CT-P13 SC and pooled IV treatment arms, the difference in the change from baseline in DAS28-CRP score was statistically significant (− 0.578; 95%: CI − 0.831, − 0.325; *p* < 0.0001), suggesting superior efficacy with CT-P13 SC. Differences in the changes from baseline in CDAI and SDAI scores were also statistically significant (− 3.502; 95% CI − 5.715, − 1.289; *p* = 0.002; and − 4.031; 95% CI − 6.385, − 1.677; *p* = 0.0008, respectively), suggesting superior efficacy with CT-P13 SC versus the pooled IV treatment arms. Similar results were obtained for the comparisons of CT-P13 SC versus CT-P13 IV, and CT-P13 SC versus reference infliximab IV (Table 3). The difference in DAS28-CRP (− 0.693) compared to reference infliximab was clinically meaningful [32].

**Table 3:** Adjusted treatment differences and odds ratios at week 30

	Covariates	Week 30			
		CT-P13 IV vs reference IFX IV	CT-P13 SC vs CT-P13 IV	CT-P13 SC vs reference IFX IV	CT-P13 SC vs IFX IV
Change from baseline in DAS28-CRP score (95% CI)	Race, baseline DAS28-CRP, anti-CPP, BMI, HAQ-DI, PADA	− 0.107 (− 0.309; 0.095), *p* = 0.3012	− 0.587 (− 0.834; − 0.34), *p* < 0.0001	− 0.693 (− 0.993; − 0.393), *p* < 0.0001	− 0.578 (− 0.831; − 0.325), *p* < 0.0001
Change from baseline in CDAI score (95% CI)	Race, baseline CDAI, anti-CPP, HAQ-DI, PADA	− 1.138 (− 2.912; 0.636), *p* = 0.2087	− 3.551 (− 5.742; − 1.36), *p* = 0.0016	− 4.690 (− 7.438; − 1.942), *p* = 0.0009	− 3.502 (− 5.715; − 1.289), *p* = 0.002
Change from baseline in SDAI score (95% CI)	Race, baseline SDAI, anti-CPP, HAQ-DI, PADA	− 1.109 (− 2.983; 0.765), *p* = 0.2462	− 4.079 (− 6.411; − 1.747), *p* = 0.0006	− 5.188 (− 8.104; − 2.272), *p* = 0.0005	− 4.031 (− 6.385; − 1.677), *p* = 0.0008
Proportion of patients achieving a EULAR good response (CRP criteria)	Race, baseline DAS28-CRP, anti-CPP, BMI, HAQ-DI, PADA	1.293 (0.913; 1.831), *p* = 0.1482	2.066 (1.389; 3.071), *p* = 0.0004	2.671 (1.722; 4.142), *p* < 0.0001	2.268 (1.556; 3.305), *p* < 0.0001
Proportion of patients achieving ACR20 response	Baseline PADA, anti-CPP, CRP, HAQ-DI, SF-36 Mental	1.129 (0.806; 1.584), *p* = 0.4803	1.442 (0.765; 2.716), *p* = 0.2580	1.628 (0.797; 3.329), *p* = 0.1818	1.439 (0.763; 2.712), *p* = 0.261
Proportion of patients achieving ACR50 response	Baseline PADA, anti-CPP, CRP, HAQ-DI, SF-36 Mental	1.133 (0.802; 1.601), *p* = 0.4777	1.601 (1.019; 2.514), *p* = 0.0414	1.814 (1.033; 3.185), *p* = 0.0384	1.597 (1.016; 2.51), *p* = 0.0429
Proportion of patients achieving ACR70 response	Baseline PADA, anti-CPP, CRP, HAQ-DI, SF-36 Mental	1.256 (0.804; 1.965), *p* = 0.3171	1.731 (1.087; 2.758), *p* = 0.0211	2.176 (1.154; 4.102), *p* = 0.0165	1.72 (1.078; 2.746), *p* = 0.0231
Proportion of patients achieving DAS-28-CRP low disease activity: DAS28-CRP ≤ 3.2	Race, baseline DAS28-CRP, anti-CPP, BMI, HAQ-DI, PADA	1.248 (0.88; 1.771), *p* = 0.2137	2.097 (1.406; 3.127), *p* = 0.0003	2.618 (1.684; 4.07), *p* < 0.0001	2.273 (1.556; 3.322), *p* < 0.0001
Proportions of patients with DAS28-CRP remission: DAS28-CRP < 2.6	Race, baseline DAS28-CRP, anti-CPP, BMI, HAQ-DI, PADA	1.218 (0.822; 1.806), *p* = 0.3258	2.308 (1.509; 3.531), *p* = 0.0001	2.812 (1.747; 4.528), *p* < 0.0001	2.481 (1.660; 3.707), *p* < 0.0001
Proportion of patients achieving CDAI low disease activity: CDAI ≤ 10.0	Race, CDAI initial, anti-CPP, HAQ-DI, PADA	1.447 (1.029; 2.035), *p* = 0.0341	2.11 (1.427; 3.120), *p* = 0.0002	3.053 (1.980; 4.708), *p* < 0.0001	2.030 (1.312; 3.142), *p* = 0.0015
Proportions of patients achieving CDAI remission: CDAI ≤ 2.8	Race, CDAI initial, anti-CPP, HAQ-DI, PADA	1.291 (0.699; 2.384), *p* = 0.4145	1.224 (0.662; 2.263), *p* = 0.52	1.58 (0.679; 3.678), *p* = 0.2891	1.213 (0.654; 2.25), *p* = 0.5412
Proportion of patients achieving SDAI low disease activity: SDAI ≤ 11.0	Race, SDAI initial, anti-CPP, HAQ-DI, PADA	1.384 (0.985; 1.944), *p* = 0.0616	2.066 (1.392; 3.066), *p* = 0.0003	2.859 (1.850; 4.418), *p* < 0.0001	2.111 (1.383; 3.223), *p* = 0.0006
Proportions of patients achieving SDAI remission: SDAI ≤ 3.3	Race, SDAI initial, anti-CPP, HAQ-DI, PADA	1.337 (0.729; 2.454), *p* = 0.3487	1.23 (0.677; 2.233), *p* = 0.4967	1.644 (0.716; 3.779), *p* = 0.2417	1.219 (0.669; 2.219), *p* = 0.5179
Proportions of patients with Boolean remission	PADA, CRP, age, BMI	1.824 (0.54; 6.165), *p* = 0.3337	2.552 (0.926; 7.034), *p* = 0.0704	4.655 (1.105; 19.622), *p* = 0.0364	2.422 (0.846; 6.938), *p* = 0.0997
Proportion of patients achieving HAQ MCID improvement	HAQ-DI, age, SF-36 mental	1.505 (1.026; 2.208), *p* = 0.0370	1.549 (0.933; 2.57), *p* = 0.0908	2.331 (1.307; 4.158), *p* = 0.0043	1.67 (1.065; 2.619), *p* = 0.0258

*Anti-CCP* anti-cyclic citrullinated peptide, *BMI* body mass index, *CDAI* Clinical Disease Activity Index, *CRP* C-reactive protein, DAS28-CRP, 28-joint Disease Activity Score based on C-reactive protein, *HAQ-DI* Health Assessment Questionnaire–Disability Index, *IFX* infliximab, *IV* intravenous, *MCID* minimal clinically important difference, *OR* odds ratio, *PADA* Patient Global Assessment of Disease Activity, *SC* subcutaneous, *SDAI* Simplified Disease Activity Index

Statistically significant differences favouring CT-P13 SC versus the pooled IV treatment arms were also observed for the majority of binary outcomes at week 30 (Table 3). The probability of achieving an ACR20 response, disease remission based on CDAI or SDAI, and Boolean remission were the only outcomes for which the effect of treatment was not statistically significantly different, although a numerical trend was observed in favour of CT-P13 SC. The odds of achieving a EULAR good response (CRP criteria) were > 2-fold higher for CT-P13 SC compared with all considered IV treatment arms. Similarly, the odds of achieving the outcome of low disease activity according to DAS28-CRP, CDAI and SDAI were also > 2-fold higher for CT-P13 SC versus all considered IV treatment arms. The ORs of patients achieving an ACR50 response ranged from 1.60 to 1.81 for the CT-P13 SC arm versus pooled IV treatment arms or reference infliximab IV; ORs of patients achieving ACR70 responses ranged from 1.72 to 2.18.

The proportion of patients achieving a clinically meaningful improvement in HAQ-DI at week 30 was statistically significantly higher with CT-P13 SC versus the pooled IV treatment arms (*p* = 0.03) (Table 3).

### Mixed treatment comparison at week 54

Altogether, 751 patients were included in the meta-regression efficacy analysis of week-54 data, which included imputed values (Table 4). From week 30 to week 54, the magnitude of the difference in the SC arm versus pooled IV treatment arms increased and the treatment difference remained statistically significant at week 54 for changes in DAS28-CRP, CDAI and SDAI, with mean differences (95% CI) estimated at − 0.876 (− 1.119, − 0.633; *p* < 0.0001), − 6.484 (− 9.026, − 3.942; *p* < 0.0001) and − 7.302 (− 9.711, − 4.893, *p* < 0.0001), respectively.

**Table 4:** Adjusted treatment differences at week 54

	Covariates	Week 54 — adjusted treatment difference, *p*-value			
		CT-P13 IV vs ref IFX IV	CT-P13 SC vs CT-P13 IV	CT-P13 SC vs Ref IFX IV	CT-P13 SC vs IFX IV
Change from baseline in DAS28-CRP score (95% CI)	Race, baseline DAS28-CRP, Anti-CPP, BMI, HAQ-DI, PADA	− 0.089 (− 0.299; 0.121), *p* = 0.4052	− 0.852 (− 1.097; − 0.607), *p* < 0.0001	− 0.941 (− 1.204; − 0.678), *p* < 0.0001	− 0.876 (− 1.119; − 0.633), *p* < 0.0001
Change from baseline in CDAI score (95% CI)	Race, baseline CDAI, HAQ-DI, PADA	− 1.014 (− 2.796; 0.768), *p* = 0.2645	− 6.283 (− 8.686; − 3.880), *p* < 0.0001	− 7.297 (− 9.831; − 4.763), *p* < 0.0001	− 6.484 (− 9.026; − 3.942), *p* < 0.0001
Change from baseline in SDAI score (95% CI)	Race, baseline SDAI, HAQ-DI, PADA	− 1.218 (− 3.119; 0.683), *p* = 0.2093	− 7.062 (− 9.318; − 4.806), *p* < 0.0001	− 8.279 (− 10.611; − 5.947), *p* < 0.0001	− 7.302 (− 9.711; − 4.893), *p* < 0.0001

*Anti-CCP* anti-cyclic citrullinated peptide, *BMI* body mass index, *CDAI* Clinical Disease Activity Index, *CRP* C-reactive protein, *DAS28-CRP* 28-joint Disease Activity Score based on C-reactive protein, *HAQ* Health Assessment Questionnaire–Disability Index, *IFX* infliximab, *IV* intravenous, *PADA* Patient Global Assessment of Disease Activity, *Ref* reference, *SC* subcutaneous, *SDAI* Simplified Disease Activity Index

## Discussion

This study compared CT-P13 SC with infliximab IV using IPD network meta-regression techniques, allowing us to compare CT-P13 SC with infliximab IV when no head-to-head trial was available. Network meta-analysis (NMA), a broader analytical framework encompassing IPD network meta-analysis, is recommended by health technology assessment agencies and scientific societies, including the National Institute for Health and Care Excellence (NICE) and the International Society for Pharmacoeconomics and Outcomes Research (ISPOR) [29, 33]. NMA is considered an ideal approach as it is capable of synthesising reliable quantitative evidence about treatment effects. The validity of NMA, particularly IPD network meta-regression, relies on the comparability of studies. In our analysis, the populations represented in the two studies had characteristics that were generally similar and the same outcome measurement scales were used. The present analysis compared the efficacy of CT-P13 SC with established IV formulations of infliximab in adult RA patients co-treated with methotrexate. Outcomes data from the CT-P13 3.5 trial appeared to favour CT-P13 SC over CT-P13 IV at week 30. Furthermore, combined analysis of data from the CT-P13 3.1 and 3.5 trials showed a statistically significant difference between the CT-P13 SC and pooled IV treatment arms for the change from baseline in DAS28-CRP, CDAI and SDAI scores; the results favoured CT-P13 SC at both the week-30 and week-54 time points. Higher response rates (e.g. ACR50/70, EULAR good response [CRP criteria]), rates of low disease activity (DAS28-CRP, CDAI and SDAI criteria), and DAS28-CRP remission rates were also observed with CT-P13 SC compared with the pooled IV treatment arms, as evaluated at the week-30 time point. Similar results were observed for the CT-P13 SC versus reference infliximab IV comparison.

Differences in the pharmacokinetic profiles of the SC and IV formulations likely account for the improved treatment outcomes observed with CT-P13 SC compared with infliximab IV in the present analysis. CT-P13 SC is administered more frequently than infliximab IV (e.g. Q2W compared with Q8W, respectively) [13]. Consequently, compared with infliximab IV, CT-P13 SC achieves a more stable steady-state serum concentration and higher trough serum concentrations (*C*_trough_) [13, 17]. For example, the median *C*_trough_ level of CT-P13 SC 120 mg in study 3.5 was 11.65 μg/mL [15]. Therefore, the *C*_trough_ with Q2W dosing of CT-P13 SC 120 mg is approximately 11 times higher than the target concentration of 1 μg/mL, which is the therapeutic threshold for the treatment of RA and the approximate *C*_trough_ achieved with Q8W dosing of infliximab IV [17, 34].

Trough serum levels of infliximab have been shown to correlate with the clinical response to infliximab treatment [35–37]. An analysis of data from the RISING study showed that in patients receiving reference infliximab IV Q8W, median trough serum infliximab concentrations were 3.0 (interquartile range 1.5–7.2), 1.1 (< 0.1–3.6) and < 0.1 (< 0.1–0.3) μg/mL for those achieving a EULAR good, moderate or no response, respectively, at week 54 [37]. The same study showed a significant association between clinical response and reduction in disease activity with higher trough serum infliximab levels (*p* < 0.001) [37]. Consistent with these findings, Wolbink and colleagues reported significantly lower trough serum infliximab levels in non-responders than responders and showed that low serum levels correlated with poor clinical improvement based on DAS28-CRP [35]. In further support of the correlation between low trough infliximab concentrations and poor clinical response, pharmacokinetic models developed for non-responsive patients in the ATTRACT trial predicted that shortening the dosing interval would have a better effect on maintaining higher trough serum levels of infliximab than increasing the dose, which in turn would increase treatment efficacy [36].

In summary, considering the totality of the evidence, it is plausible that improved efficacy of CT-P13 SC is due to achieving higher *C*_trough_ levels through more frequent administration, compared with infliximab IV. Furthermore, there were no clinically meaningful differences between the safety profiles of CT-P13 SC and CT-P13 IV in study 3.5 [16], suggesting that higher trough serum concentrations observed with CT-P13 SC do not appear to be associated with an increased risk of adverse events.

Several limitations should be taken into consideration when interpreting the results of the present analysis. First, data were obtained from only two trials, and data from other infliximab RCTs were not included in the analysis. It was deemed that the integration of aggregate data from infliximab RCTs without CT-P13 SC would not add substantial value to this analysis, although the inclusion of more studies would arguably better account for variability in outcomes of infliximab IV between studies. Furthermore, the comparison of data from earlier infliximab trials with data from more recently conducted trials, such as study 3.1, may be inappropriate due to differences in disease duration and progression in the enrolled populations [17, 20, 38, 39]. A second limitation was the use of imputation for missing values (i.e. week-54 data), which relies on the assumption that the evolution of clinical scores after week 30 in patients who remained on CT-P13 IV in the 3.5 trial would be comparable to that observed in study 3.1. However, the uncertainty associated with extrapolation was fully accounted for in the reported 95% CIs, based on multiple imputation. Moreover, due to the high uncertainty associated with predicting binary outcomes at week 54, mixed treatment comparisons were reported as continuous variables only [33]. Thirdly, radiological outcomes were not assessed in the present meta-regression, as structural damage was not evaluated in the CT-P13 3.5 trial; however, other studies provide evidence that infliximab is effective in reducing radiological evidence of synovitis and erosions in the long term [40]. Finally, as the focus of the present analysis was the comparative efficacy of CT-P13 SC and infliximab IV, safety endpoints were not analysed. However, safety data from study 3.5 suggest that the safety profile of the CT-P13 SC is at least similar, or even favourable, compared with CT-P13 IV; for example, the proportions of patients experiencing treatment-emergent adverse events (TEAEs), serious TEAEs, TEAEs causing drug discontinuation, and infection were numerically lower in the SC arm, compared with the IV treatment arm, up to week 54 [16] (Additional file 1, Table S4). Finally, long-term data regarding CT-P13 SC safety and efficacy are limited and should be collected in future studies.

## Conclusions

This meta-regression of IPD from two randomised trials showed that CT-P13 SC was associated with greater improvements in DAS28-CRP, CDAI and SDAI scores; higher proportions of patients achieving ACR and EULAR responses; low disease activity; and clinically meaningful improvements in functional disability, compared with CT-P13 IV and reference infliximab IV. Thus, CT-P13 SC may be a valuable alternative to infliximab IV.

## Supplementary Information

**Additional file 1: Table S1.** List of variables used as potential covariates. **Table S2.** Pearson correlation between baseline parameters. **Table S3.** Prediction models in Remsima IV treatment arm (Study 3.1). **Table S4.** Comparison of safety outcomes in Studies 3.5 and 3.1.

## Data Availability

The datasets used and/or analysed during the current study are available from the corresponding author on reasonable request.

## Abbreviations

ACR: American College of Rheumatology
AE: Adverse event
anti-CCP: Anti-cyclic citrullinated peptide
bDMARD: Biologic synthetic disease-modifying antirheumatic drug
BMI: Body mass index
CDAI: Clinical Disease Activity Index
CI: Confidence interval
CRP: C-reactive protein
*C*_trough_: Trough serum concentration
DAS28-CRP: 28-Joint Disease Activity Score based on C-reactive protein
DAS28-ESR: 28-Joint Disease Activity Score based on erythrocyte sedimentation rate
DMARD: Disease-modifying antirheumatic drug
ESR: Erythrocyte sedimentation rate
EU: European Union
EULAR: European League Against Rheumatism
HAQ-DI: Health Assessment Questionnaire–Disability Index
HRQoL: Health-related quality of life
IFX: Infliximab
IPD: Individual patient data
ISPOR: International Society for Pharmacoeconomics and Outcomes Research
IV: Intravenous
MCID: Minimal clinically important difference
MTX: Methotrexate
NICE: National Institute for Health and Care Excellence
NMA: Network meta-analysis
OR: Odds ratio
PADA: Patient Global Assessment of Disease Activity
PAPA: Patient Assessment of Pain
PHDA: Physician Assessment of Disease Activity
PK: Pharmacokinetic(s)
Q#W: Every # weeks
RA: Rheumatoid arthritis
RCT: Randomised controlled trial
SC: Subcutaneous
SD: Standard deviation
SDAI: Simplified Disease Activity Index
SF-36: 36-Item Short-Form Health Survey
SJC28: Swollen 28-Joint Count
TEAE: Treatment-emergent adverse event
TESAE: Treatment-emergent serious adverse event
TJC28: Tender 28-Joint Count
TNFi: Tumour necrosis factor inhibitor
tsDMARD: Targeted synthetic disease-modifying antirheumatic drug
