# Supplementary material for: Comparative efficacy of subcutaneous (CT-P13) and intravenous infliximab in adult patients with rheumatoid arthritis: a network meta-regression of individual patient data from two randomised trials
Source: Arthritis Res Ther. 2021 Apr 16;23:119. doi: 10.1186/s13075-021-02487-x (PMC8051052; doi:10.1186/s13075-021-02487-x)
Supplement: Supplementary file 1 — Additional file 1: Table S1. List of variables used as potential covariates. Table S2. Pearson correlation between baseline parameters. Table S3. Prediction models in Remsima IV treatment arm (Study 3.1). Table S4. Comparison of safety outcomes in Studies 3.5 and 3.1. [file 13075_2021_2487_MOESM1_ESM.pdf]

Additional file 1

Comparative efficacy of subcutaneous (CT-P13) and intravenous infliximab in adult patients with rheumatoid arthritis: a network meta-regression of individual patient data from two randomised trials

Bernard Combe, Yannick Allanore, Rieke Alten, Roberto Caporali, Patrick Durez, Florenzo Iannone, Michael T. Nurmohamed, Mondher Toumi, Sang Joon Lee, Taek Sang Kwon, Jiwon Noh, Gahee Park and Dae Hyun Yoo

Correspondence to: Dae Hyun Yoo, Department of Rheumatology, Hanyang University Hospital for Rheumatic Diseases, 222-1 Wangsimni-Ro, Seongdong-Gu, Seoul 04763, Republic of Korea; dhyoo@hanyang.ac.kr

Table S1 List of variables used as potential covariates

- **Demographic characteristics**
  - Age (≤50 / >50)
  - Sex (female / male)
  - Weight*
  - BMI*
  - Race (Caucasian White / Asian / Other)
  - Region (Europe / Asia / Latin America)
- **Clinical characteristics**
  - CRP (≤0.6 / >0.6)
  - ESR*
  - Anti-CCP antibody (positive / negative) as defined in the CT-P13 3.5 study
  - TJC (28 joints)*
  - SJC (28 joints)*
  - CDAI*
  - SDAI*
  - DAS28-CRP*
  - DAS28-ESR*
  - HAQ*
  - SF-36 (Physical / Mental)*
  - Patient assessment of pain*
  - Patient global assessment of disease activity*
  - Physician global assessment of disease activity*

*Continuous variable

Anti-CCP, anti-cyclic citrullinated peptide; BMI, body mass index; CDAI, Clinical Disease Activity Index; CRP, C-reactive protein; DAS28-CRP, 28-joint Disease Activity Score based on C-reactive protein; DAS28-ESR, 28-joint Disease Activity Score based on erythrocyte sedimentation rate; ESR, erythrocyte sedimentation rate; HAQ, Health Assessment Questionnaire; SDAI, Simplified Disease Activity Index; SF-36, 36-item Short-Form Health Survey; SJC, swollen joint count; TJC, tender joint count

Table S2 Pearson correlation between baseline parameters

	Age	BMI	Weight	CDAI	SDAI	DAS28-CRP	DAS28-ESR	ESR	CRP	SF-36 mental	SF-36 physical	PADA	PHDA	PAPA	SJC28	TJC28
Age	1.000															
BMI	0.258	1.000														
Weight	0.181	0.856	1.000													
CDAI	0.075	0.081	0.066	1.000												
SDAI	0.070	0.067	0.054	0.974	1.000											
DAS28-CRP	0.064	0.084	0.071	0.871	0.932	1.000										
DAS28-ESR	0.099	0.064	0.029	0.887	0.899	0.880	1.000									
ESR	0.054	−0.045	−0.088	0.081	0.176	0.262	0.462	1.000								
CRP	−0.009	−0.050	−0.045	0.119	0.340	0.471	0.257	0.433	1.000							
SF-36 mental	−0.084	−0.066	−0.022	−0.235	−0.244	−0.271	−0.263	−0.057	−0.084	1.000						
SF-36 physical	−0.051	−0.011	−0.004	−0.317	−0.342	−0.390	−0.367	−0.149	−0.181	0.180	1.000					
PADA	0.093	0.043	0.020	0.469	0.485	0.571	0.552	0.129	0.174	−0.328	−0.439	1.000				
PHDA	0.118	0.045	0.036	0.502	0.514	0.505	0.493	0.122	0.166	−0.284	−0.348	0.709	1.000			
PAPA	0.068	0.126	0.146	0.442	0.461	0.526	0.496	0.085	0.197	−0.369	−0.501	0.854	0.663	1.000		
SJC28	−0.007	0.048	0.051	0.844	0.822	0.678	0.692	0.086	0.100	−0.098	−0.187	0.178	0.236	0.224	1.000	
TJC28	0.088	0.087	0.068	0.905	0.868	0.771	0.799	0.015	0.050	−0.190	−0.225	0.259	0.288	0.242	0.645	1.000
HAQ-DI	0.132	0.085	0.004	0.378	0.395	0.449	0.435	0.149	0.154	−0.453	−0.623	0.504	0.428	0.514	0.212	0.278

BMI, body mass index; CDAI, Clinical Disease Activity Index; CRP, C-reactive protein; DAS28-CRP, 28-joint Disease Activity Score based on C-reactive protein; DAS28-ESR, 28-joint Disease Activity Score based on erythrocyte sedimentation rate; ESR, erythrocyte sedimentation rate; HAQ-DI, Health Assessment Questionnaire–Disability Index; PADA, Patient Global Assessment of Disease Activity; PAPA, Patient Assessment of Pain; PHDA, Physician Assessment of Disease Activity; SDAI, Simplified Disease Activity Index; SF-36, 36-item Short-Form Health Survey; SJC28, Swollen 28-Joint Count; TJC28, Tender 28-Joint Count

Table S3 Prediction models in Remsima IV treatment arm (Study 3.1)

Outcome	Variables				Fit statistics
		Coeff.	SD	*p*-value	R^2
CDAI change from baseline at Week 54	Intercept	−5.98	3.79	0.1163	0.61
	CDAI at baseline	−0.41	0.07	<0.0001	
	change of CDAI from baseline at Week 30	0.52	0.06	<0.0001	
	HAQ-DI at Week 30	4.04	1.14	0.0005	
	SF-36 mental at Week 30	0.14	0.06	0.0255	
DAS28-CRP change from baseline at Week 54	Intercept	−0.47	0.58	0.4169	0.48
	DAS28-CRP at baseline	−0.25	0.09	0.0056	
	DAS28-CRP change at Week 30	0.6	0.06	<0.0001	
	HAQ-DI at Week 30	0.35	0.14	0.0102	
	SF-36 mental at Week 30	0.02	0.01	0.0283	
SDAI change from baseline of at Week 54	Intercept	−4.85	4.12	0.2405	0.61
	SDAI at baseline	−0.35	0.07	<0.0001	
	SDAI change from baseline at Week 30	0.55	0.06	<0.0001	
	HAQ-DI at Week 30	3.89	1.2	0.0014	
	SF-36 mental at Week 30	0.15	0.07	0.0201	
	ESR at baseline	−0.06	0.03	0.0397	

CDAI, Clinical Disease Activity Index; CRP, C-reactive protein; DAS28-CRP, 28-joint Disease Activity Score based on C-reactive protein; ESR, erythrocyte sedimentation rate; HAQ-DI, Health Assessment Questionnaire–Disability Index; IV, intravenous; SD, standard deviation; SDAI, Simplified Disease Activity Index; SF-36, 36-item Short-Form Health Survey

Table S4 Comparison of safety outcomes in Studies 3.5 and 3.1

	Study CT-P13 3.5[1]		PLANETRA (Study CT-P13 3.1)		
Dosage	CT-P13 SC 120 mg Q2W	CT-P13 IV 3 mg/kg Q8W – SC 120 mg Q2W[2]	CT-P13 IV 3 mg/kg Q8W	Reference IFX IV 3 mg/kg Q8W	Pooled CT-P13 IV/ Reference IFX IV 3 mg/kg Q8W
Patients using immunosuppressant %	100%	100%	100%	100%	100%
Patients, *n*	168	175	302	300	602
Patients with ≥1 event, *n* (%)					
TEAE	92 (54.8)	117 (66.9)	213 (70.5)	211 (70.3)	424 (70.4)
TESAE	6 (3.6)	13 (7.4)	42 (13.9)	31 (10.3)	73 (12.1)
Infection	49 (29.2)	60 (34.3)	127 (42.1)	137 (45.7)	264 (43.9)
Serious infection	3 (1.8)	1 (0.6)	13 (4.3)	7 (2.3)	20 (3.3)
TEAE leading to study drug discontinuation	6 (3.6)	20 (8.0)	33 (10.9)	47 (!5.7)	80 (13.3)

Note: For Study CT-P13 3.5, the safety results are from the Maintenance Phase (from Week 6 onwards)

[1]Patients in Bulgaria, Poland, and Russia received CT-P13 SC up to Week 64

[2]Patients in the IV treatment arm switched to CT-P13 SC treatment at Week 30

IFX, infliximab; IV, intravenous; Q#W, every # weeks; SC, subcutaneous; TEAE, treatment-emergent adverse event; TESAE, treatment-emergent serious adverse event
